# Antiviral Drugs for Treatment of Patients Infected with Pandemic (H1N1) 2009 Virus

**DOI:** 10.3201/eid1511.090720

**Published:** 2009-11

**Authors:** David M. Hartley, Noele P. Nelson, Eli N. Perencevich

**Affiliations:** Georgetown University Medical Center, Washington, DC, USA (D.M. Hartley, N.P. Nelson); University of Maryland Medical Center, Baltimore, Maryland, USA (E.N. Perencevich)

**Keywords:** Influenza, pandemic, H1N1, antimicrobial resistance, antiviral drugs, viruses, expedited, letter

**To the Editor:** The emergence of influenza A pandemic (H1N1) 2009 virus in North America and associated illness and death suggest that humanity faces a dangerous threat. Viruses isolated from a sample of patients with confirmed cases in early phases of the outbreak demonstrated resistance to amantadine and rimantadine. At present, circulating viruses appear to be largely susceptible to the neuraminidase inhibitors oseltamivir and zanamivir, although oseltamivir resistance has been observed in recent cases in Europe, Asia, and North America (*1*). More recently, pandemic (H1N1) 2009 virus resistance to oseltamivir emerged during treatment of 2 immunosuppressed patients in the United States. Such cases demonstrate that oseltamivir resistance can emerge in infected persons treated with oseltamivir. To date, all isolates tested have been susceptible to zanamivir.

Vaccines are being deployed in some well-resourced countries but are generally not available to the public. It appears that little if any protection is offered from previous seasonal influenza vaccines. In the spring of 1918, epidemiologic observations indicated the likely emergence and spread of another influenza virus (H1N1) that caused few deaths. However, later that year, transmission resurged and was associated in 2 waves with increased illness and deaths. We cannot predict whether the 2009 pathogen will follow a similar temporal pattern and evolve toward increased virulence. Even if vaccine development and delivery could be achieved within 6 months, an aggressive schedule, large supplies of vaccine against pandemic (H1N1) 2009 may not be available until late 2009.

Antiviral drugs are used to treat patients with strongly suspected or confirmed influenza. However, until a vaccine is available, specific protection by pharmaceutical products is limited to antiviral drugs. Nonpharmaceutical interventions are also available for prevention. Some governments and organizations are taking steps that would enable mass administration of these drugs (*2*). This administration may prove problematic. A recent study showed that schoolchildren may incompletely adhere to oseltamivir prophylaxis instructions (*3*). If other groups are given oseltamivir prophylaxis, they cannot necessarily be expected to follow administration guidelines; compliance with taking the recommended number of doses at appropriate times is difficult to enforce. Moreover, even when compliance is high, oseltamivir prophylaxis may fail (*4*).

The first viable oseltamivir-resistant human influenza viruses (H1N1) emerged and became prevalent in the United States and Europe in the 2007–08 influenza season, and prevalence of such viruses has continued in 2009. The potential for overuse of antiviral drugs, especially oseltamivir, to select for existing antiviral drug-resistant strains is unknown. Ecologic studies suggest a lack of association between prevalence of oseltamivir use and prevalence of oseltamivir resistance (*5*). However, examination of seasonal influenza virus isolates obtained before introduction of oseltamivir showed an absence of resistance (*6*), leading some to conclude that antiviral monotherapy leads to selection pressure for resistance (*7*). Regardless of origin of resistance, recent seasonal influenza viruses (H1N1) of the A/Brisbane/57/2007 lineage from around the world display such resistance.

A similar resistance pattern could occur with pandemic (H1N1) 2009 virus. Regardless of the mutational mechanism for antiviral drug resistance, mass use of antiviral drugs could potentially lead to selection pressure for drug-resistant viruses (*7*). Experience with seasonal influenza demonstrated the fitness of some oseltamivir-resistant strains (*8*). Moreover, modeling studies suggest that antiviral-resistant strains may spread rapidly and markedly affect pandemic outcomes (*9*).

What are we to do? Until a vaccine is available, combination antiviral therapy and rapid diagnostic testing may be needed (*7*). Given the recently described low sensitivity of currently available rapid tests, applying such assays to all patients is problematic (*10*). If rapid testing has a role, it should be used in testing persons at highest risk for developing influenza complications. However, early empiric therapy based on clinical manifestations and knowledge of circulating strains is likely more appropriate than reliance on tests with low sensitivity. Updated guidelines recently issued by the World Health Organization (www.who.int/csr/resources/publications/swineflu/h1n1_guidelines_pharmaceutical_mngt.pdf) and the Centers for Disease Control and Prevention (www.cdc.gov/h1n1flu/recommendations.htm) for prophylaxis should be followed to keep resistance in check and save the lives of patients.

A widely administered protective vaccine is needed to prevent transmission and infection and preserve the efficacy of antiviral agents. Indiscriminant administration of these agents could support proliferation of antiviral resistance in pandemic (H1N1) 2009 virus or an evolved variant. Appropriate use of antiviral chemotherapy is complex. Identifying the groups at high risk for serious illness for drug therapy and appropriate antiviral therapy in situations of co-circulation of seasonal and pandemic (H1N1) viruses with various susceptibility patterns needs elucidation. Without clear evidence-based guidance, a global public health disaster could occur if pandemic (H1N1) 2009 reemerges later this year with higher virulence or widespread antiviral drug resistance.
